# Application of a self-assembling peptide matrix prevents esophageal stricture after circumferential endoscopic submucosal dissection in a pig model

**DOI:** 10.1371/journal.pone.0212362

**Published:** 2019-03-12

**Authors:** Sarra Oumrani, Maximilien Barret, Benoit Bordaçahar, Frédéric Beuvon, Guillaume Hochart, Aurélie Pagnon-Minot, Romain Coriat, Frédéric Batteux, Frédéric Prat

**Affiliations:** 1 Department of Gastroenterology, Cochin Hospital, APHP, Paris, France; 2 INSERM Unit 1016, Paris, France; 3 Pathology Department, Cochin Hospital, APHP, Paris, France; 4 Imabiotech, Loos, France; 5 Novotec, Bron, France; 6 Department of Immunology, Cochin Hospital, APHP, Paris, France; University Hospital Hamburg Eppendorf, GERMANY

## Abstract

**Introduction:**

Circumferential endoscopic submucosal dissection (ESD) allows to treat large esophageal superficial neoplasms, however with a high occurrence of severe esophageal strictures. In a previous work, we demonstrated that the application of a prototype of self-assembling peptide (SAP) matrix on esophageal wounds after a circumferential-ESD delayed the onset of esophageal stricture in a porcine model. The aim of this work was to consolidate these results using the commercialized version of this SAP matrix currently used as a hemostatic agent.

**Animals and methods:**

Eleven pigs underwent a 5 cm-long circumferential esophageal ESD under general anesthesia. Five pigs were used as a control group and six were treated with the SAP. In the experimental group, 3.5 mL of the SAP matrix were immediately applied on the ESD wound. Stricture rates and esophageal diameter were assessed at day 14 by endoscopy and esophagram, followed by necropsy and histological measurements of inflammation and fibrosis in the esophageal wall.

**Results:**

At day 14, two animals in the treated group had an esophageal stricture without any symptom, while all animals in the control group had regurgitations and an esophageal stricture (33 vs. 100%, p = 0.045). In the treated group, the mean esophageal diameter at day 14 was 9.5 ± 1 mm vs. 4 ± 0.6 mm in the control group (p = 0.004). Histologically, the neoepithelium was longer in the SAP treated group vs. the control (3075 μm vs. 1155μm, p = 0.014). On immunohistochemistry, the expression of alpha smooth muscle actin was lower in the treated vs. control group.

**Conclusion:**

Apposition of a self-assembling peptide matrix immediately after a circumferential esophageal ESD reduced by 67% the occurrence of a stricture at day 14, by promoting reepithelialization of the resected area.

## Introduction

With the increasing number of endoscopic resection procedures for the treatment of early esophageal cancer and Barrett’s esophagus, [1] and the expansion of endoscopic submucosal dissection in the esophagus [2], preventing the formation of an esophageal stricture during the wound healing process has become a major clinical challenge. Indeed, post endoscopic esophageal strictures occur in up to 90% of patients with mucosal defects exceeding three fourths of the esophageal circumference [3].

A potential strategy would be to use biological wound dressing on the exposed submucosa to prevent excessive scar tissue formation. A self-assembling peptide (SAP) formed by an aqueous solution of a short synthetic peptide RADA-16 (4 Arg-Ala-Asp-Ala) has been developed and is commercialized as a hemostatic agent (PuraStat, 3-D Matrix Europe SAS, Caluire-et-Cuire, France). Promising effects of an initial version of this matrix at a pH = 3.1 were demonstrated in an animal model of post endoscopic esophageal stricture [4]. Our aim is now to assess PuraStat, the commercially available version of the SAP which is at pH = 2, as a wound dressing for the prevention of esophageal stricture after circumferential ESD.

## Material and methods

### Ethical aspects

This protocol received approval from the animal ethical committee of the Paris Descartes University and from the department of national education and research (reference number 15–034).

### Study design

It was a retrospective comparison of two groups of pigs which were both managed prospectively by our team. All animals underwent circumferential ESD in a treated group (n = 6) with apposition of the SAP matrix right after the endoscopic resection and a control group (n = 6). We have two groups with different number of animals because we oversize of 10% our animal study, given a 10% risk of death by esophageal perforation following the endoscopic procedure. In the control group, one pig died of an esophageal perforation the same day of the procedure and therefore was excluded from the analysis.

### Description of procedure and follow-up

The animals all came from the same farm (Neuve-maison, FRANCE) and were accommodated at our facility 48 hours before the procedure. They were *Sus scrofa domesticus* species. Details on ESD procedure, animal care and follow-up are provided in a paper from Barret et al. [5]. Briefly, we performed a circumferential ESD (CSED) in the distal third of the esophagus of a 5-cm length, 5 cm above the esophagogastric junction in 11 swine under general anesthesia. Then, immediately after the resection, 3.5 ml of the SAP matrix was applied on the entire resection wound for the 6 treated animals using a dedicated 2.8 mm specific catheter (PuraStat Nozzle System type E, Top Corporation, MPS Medical Product Service GmbH, Germany). The entire wound was covered by the SAP matrix. For all animals, the SAP did not clear out at the end of the application and we didn’t have to use any associated method to fix it to the wound.

After the procedure, pigs were not given any food or liquids for 24 hours so that the SAP remained in place. They were then given a liquid meal (made of oral nutritional complements) for 3 days. After 7 days, they were given semi-liquid meals made of moist beans.

Daily clinical examination and weekly endoscopic follow-up was performed with assessment of the esophageal wound and diameter -estimated with an open 7 mm biopsy forceps-, as well as a barium esophagram at day 14. All animals were euthanized with 100 mg/kg intravenous injection of pentobarbital (Dolethal, Vétoquinol, Paris, France) at day 14 after the CESD and underwent necropsy with *en bloc* esophagectomy.

### Histological analysis and immunohistochemistry

After gross morphological examination, specimens were fixed in 10% buffered formalin, embedded in paraffin, and processed into 5-μm-thick sections. Slides were stained with hematoxylin eosin and saffron (HES) and digitized. A senior pathologist, blinded from the treatment group, analyzed all slides. On digitized representative slides, we measured fibrosis, granulation tissue, and length of the neoepithelium (characterized by round immature epithelial cells without native epithelial crest and chorionic papillae or parakeratosis). The inflammatory cell infiltrate of the granulation tissue was characterized as acute (predominance of polynuclear cells and high cell density) or chronic (predominance of lymphocytes or plasmocytes, low cell density). Immunohistochemical staining for Type I (Novotec, ref. 20191) and Type III Collagens (Novotec, ref. 20341) and alpha-SMA (Dako, M0851) was performed. Antigen-antibodies complexes are revealed by tetrahydrochloride diaminobenzidine (DAB, Dako, K3468) and cells are slightly counterstained with Mayer’s hematoxylin. Pictures were taken with a digital camera (LAS Version 4) and edited by image analysis software (Adobe, PhotoShop cc2015.5). Semi-quantitative signals were determined ranging from 1 to 3.

### Preliminary study: MALDI (matrix-assisted laser desorption ionization) spectrometry imaging

In a preliminary study, aiming to assess the stability of the SAP on the esophageal wounds, we performed two esophageal submucosal dissections in a pig and applied 1ml PuraStat on one of the two wounds. At day 1, the animal was euthanized and an esophagectomy was performed. The samples were frozen in dry ice and send to Imabiotech (Lille, France) for the MALDI-imaging spectrometry. 10μm-thick sections were prepared and collected on ITO (Indium Tin Oxide) glass slides for MALDI analysis or on Superfrost slides for HE staining. DHB (2,5-Dihydroxybenzoic acid) MALDI matrix (40mg/mL in methanol/H_2_O 0.1% TFA (trifluoroacetic acid) 1:1 (v/v)) was sprayed on the tissue sections using the automatic TM Sprayer device (HTX Imaging, Chapel Hill, North Carolina). CASI (Continuous Accumulation of Selected Ions) positive mode centered on *m/z* 1713 +/- 40 Da mass ranges was selected for imaging PuraStat in the sections at 50μm spatial resolution in selected regions by 7T-MALDI-FTICR SolariX mass spectrometer (Bruker Daltonics, Bremen, Germany). FTMS (Fourier transform mass spectrometer) Control 2.0 and FlexImaging 4.1 software packages (Bruker Daltonics, Bremen, Germany) were used to control the mass spectrometer and set imaging parameters. Multimaging (ImaBiotech, Lille, France) was used for the data analysis and treatment of the images with overlays.

### Endpoints

The primary endpoint was the occurrence of a symptomatic esophageal stricture at day 14 after circumferential ESD. Esophageal stricture was defined as the impossibility to cross the scarred area with a 10-mm gastroscope. Secondary endpoints were: weight variation between day 0 and day 14, esophageal diameter assessed endoscopically at day 14, stricture index at day 14 (defined by the ratio between the narrowest and the widest esophageal diameter proximal to the stricture on esophagram at day 14), thickness of esophageal fibrosis, granulation tissue layers and length of the neoepithelium on histological slides, expression of alfa smooth muscle actin and type I and III collagen.

### Statistical analysis

Statistical analysis was performed using SPSS version 23.0. Continuous data are expressed as median values and interquartile range (IQR) and compared with an unpaired t-test. Categorical data are expressed as percentages and compared with a Fisher’s exact test. A p-value < 0.05 indicates statistical significance.

## Results

### Preliminary study: MALDI-imaging spectrometry

**Fig 1:**
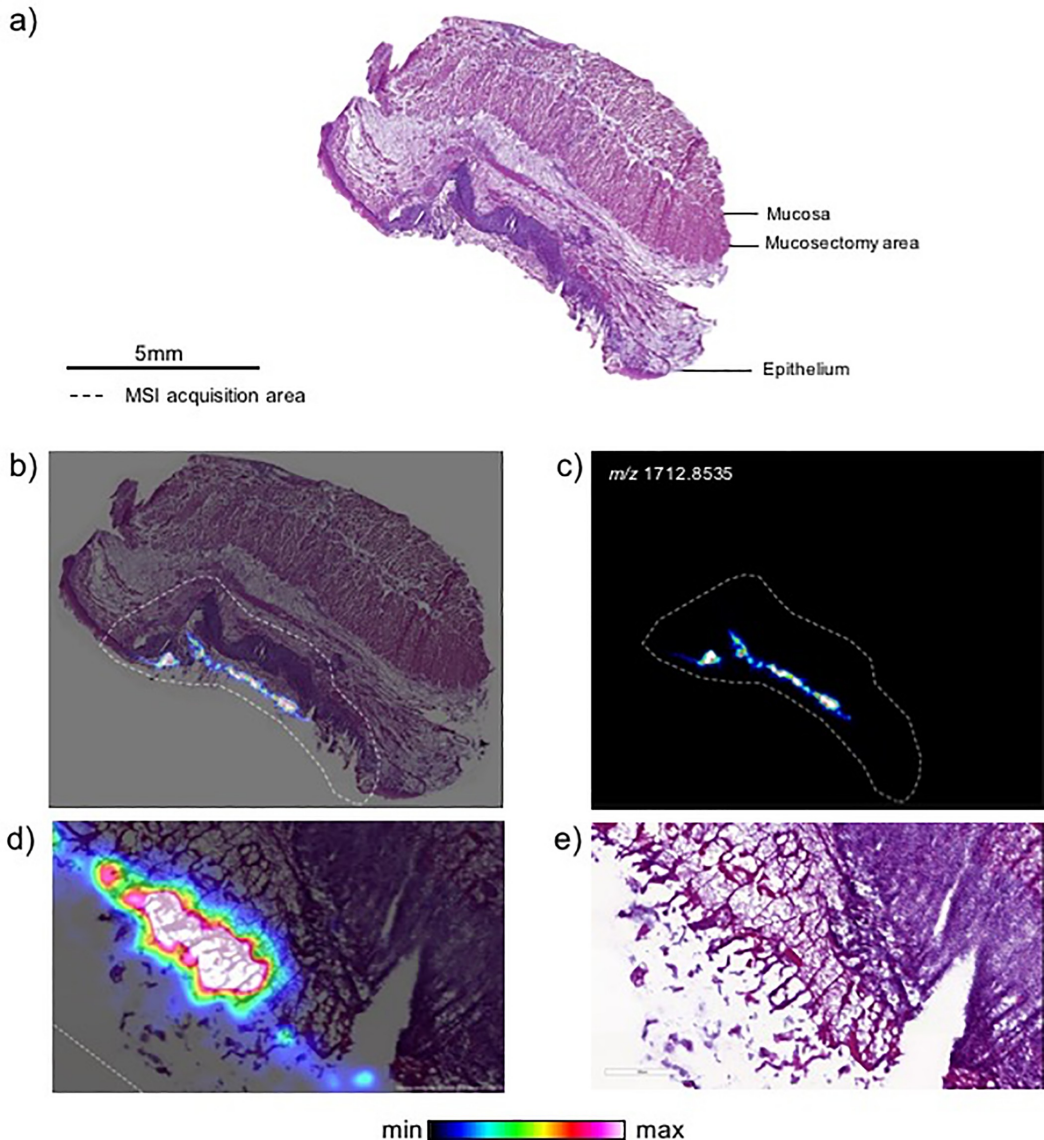
Distribution of PuraStat with MALDI spectrometry imaging in treated pig esophagus section at day 1. a) Hematoxylin & Eosin (HE) staining of a serial section; b) Overlay of the PuraStat distribution and the HE staining, c) PuraStat molecular distribution only; d) Zoom in the overlay; e) Zoom in the HE staining only.

The self-assembled peptide RADA16 was detected on the treated resection wound and not on the control wound. The distribution in the treated tissue and the overlay of the molecular image of the RADA16 distribution and the HE staining are presented in Fig 1.

### Clinical and endoscopic outcomes after SAP application

Five pigs were included in the control group and six in the group treated with the SAP matrix. Mean ± SD procedure duration was 40 ± 8 min, and no adverse event occurred.

Median weight in the treated group 29.75 kg and 31.6 kg in the control group (p = 0.358).

At day 7, there were no esophageal stricture in any animal. At day 14, two animals in the SAP treated group had an esophageal stricture without any symptom while all animals in the control group had a symptomatic esophageal stricture (33 *vs*. 100%, p = 0.045). In the SAP treated group, the median esophageal diameter at day 14 was 9.5 ± 1 mm *vs*. 4 ± 0.6 mm in the SAP treated *vs*. control group (p = 0.004). Median esophageal stricture indexes assessed on barium esophagogram were 0.53 *vs*. 0.26 in the SAP treated *vs*. control group (p = 0,000413) (Table 1).

**Table 1:** Clinical and endoscopic outcomes in the SAP treated and control groups at day 14 after esophageal circumferential endoscopic submucosal dissection.

	Control group (n = 5)	SAP treated group (n = 6)	p value
**Clinical outcomes**			
- Weight variation (kg)—median (IQR)	-3.8 (-4.6; -0.1)	1.6 (1.4; 3.5)	p = 0.017 IC95% [1.31; 9.05]
- Stricture related symptoms* (n)	5	0	p = 0.002
**Endoscopic outcomes**			
- Esophageal stricture (n)	5	2	p = 0.045
- Esophageal diameter (mm)–median (IQR)	4 (3; 4)	9.5 (8.2; 10)	p = 0.004 IC95% [4.5; 6.6]
**Esophagram findings**			
- Stricture index—median (IQR)	0.26 (0,17; 0.3)	0.53 (0.5; 0.65)	p = 0.000413 IC95% [0.20; 0.47]

*regurgitations, aphagia

SAP: self-assembling peptide

### Histological findings after SAP application

The thickness of the inflammatory cell infiltrate was 694 (IQR 558–880) μm *vs*. 122 (IQR 118–140) μm in the SAP *vs*. control group (p = 0.004). In both groups, the majority of the cells involved were acute inflammatory cells. The thickness of esophageal fibrosis was 342.5 (IQR 244–490) μm *vs*. 170 (IQR 139–202) μm (p = 0.052) and the length of the neoepithelium was 3075 (2450–3835) μm *vs*. 1155 (737–1616) μm (p = 0.014) for the SAP treated *vs*. control group, respectively. Pathology features are summarized in Table 2.

**Table 2:** Pathology findings in the SAP treated and control groups at day 14 after esophageal circumferential endoscopic submucosal dissection.

	Control group (n = 5)	SAP-treated group (n = 6)	p value
**Inflammatory cell infiltrate (μm)–median (IQR)**	122 (118–140)	694 (558–880)	0.004
**Type of inflammatory cells**			
- Acute	4	5	1
- Chronic	1	1	1
**Fibrosis thickness (μm)–median (IQR)**	170 (139–202)	342.5 (244–490)	0.052
**Neoepithelium length (μm)—median (IQR)**	1155 (737–1616)	3075 (2450–3835)	0.014

SAP: self-assembling peptide

**Fig 2:**
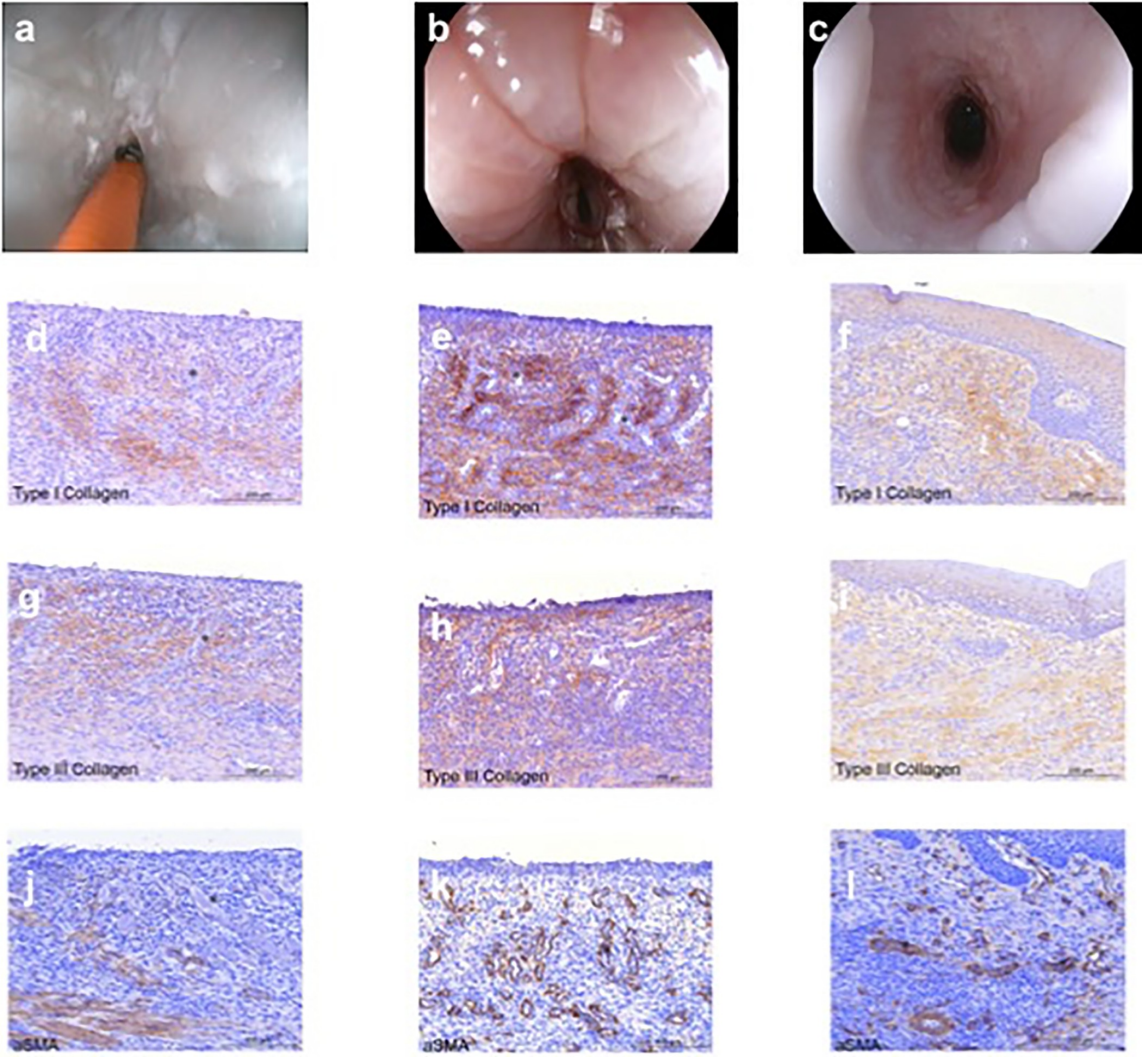
Endoscopic pictures for a control pig (a), a treated pig who developed an endoscopic stenosis (b) and a treated pig without stenosis at day 14 (c) and immunohistochemistry labelling of Collagen I (d, g, j), Collagen III (e, h, k) and Alpha-SMA (f, I, l) for each group.

Mean signals for Alpha-SMA, Collagen I and Collagen III were 1.33 ± 0.52 *vs*. 2.4 ± 0.89 (N/S, p = 0.08); 2.33 ± 0.52 *vs*. 2.4 ± 0.55 (N/S); 1.17 ± 0.41 *vs*. 1.6 ± 0.55 (N/S) in the SAP treated group and the control group respectively. Frames are summarized in Fig 2.

## Discussion

A large number of treatment options have been evaluated to prevent the formation of an esophageal stricture after a circumferential or an extensive ESD [6]. Currently, among them, corticosteroids, either orally administered or injected in the resection wound, are one of the most effective treatment [7,8]. However, some complications have been described following the corticosteroids injection such as peri-esophageal abscess [9], and severe infectious morbidity has also been reported after systemic administration of steroids in this setting [10]. Previous studies evaluated anti-inflammatory agents such as Pentoxifylline associated with interferon-alpha, N-acetylcysteine and showed limited effects [11,12]. Other options include wound-covering agents, such as acellular membranes or biological wound dressings such as a self assembling peptide matrix [6].

Kumar et al. used a synthetic peptide hydrogel for 3D co culture with esophageal cells [13]. Although this *in vitro* situation is different from esophageal stricture, it suggests the role of peptide gels in the healing process. The SAP matrix that we used in this work is currently commercialized for surgical or endoscopic hemostasis. Furthermore, several studies found the SAP to also improve the wound healing process of the digestive mucosa [14] and the skin [15,16].

Based on clinical, endoscopic and radiologic data, we demonstrated the efficacy of the SAP matrix to reduce the rate of esophageal stricture at day 14 after a circumferential ESD on a pig model.

An explanation is brought by pathology and immunohistochemistry. SAP-treated pigs had a thicker inflammatory infiltrate and a higher Collagen I/III ratio compared to controls. Conversely, fibrosis thickness was higher in SAP treated animals than in controls but alpha-SMA staining was lower, although this results were not significant. Finally, the length of the neoepithelium was significantly increased in the SAP treated group as compared to controls. Our hypothesis is that the SAP limits the scar tissue formation, possibly by promoting reepithelialization.

Studies on corticosteroids or amniotic membrane grafts have shown a reduction in the density of myofibroblasts in the esophagus during the wound healing to be associated with an effective stricture prevention [17,18]. In the healing process after an esophageal mucosal injury, myofibroblasts take part when epithelial cells migrate from the edge of the wound and proliferate, promoted by angiogenesis. At this time, myofibroblasts may promote the development of the stricture.

We hypothesized that only one application immediately after the ESD procedure could be sufficient. Indeed, previous studies in the digestive tract suggested that the SAP matrix could be found until 6 days after first application by histology observation [14]. We performed a SAP detection found at 24 hours after application with a MALDI (matrix-assisted laser desorption ionization) spectrometry imaging on the surface of the wound after an ESD procedure, since the endoscopic visualization of the SAP is extremely difficult, as is the identification of SAP on pathology slides. Although this new technique of detection was performed on only one pig, the SAP repartition appears homogeneous on the whole surface of the wound where it has been applied. To our knowledge, this is the first identification of the SAP using the technique of MALDI-imaging spectrometry and could be used in the future to detect precisely how long the SAP can stay on the wound.

The main limitations of our study include the small number of animals, and the absence of long term follow-up. Indeed, we chose to sacrifice the SAP treated pigs at day 14 in order to have histologically comparable data between both groups. Further study with long term surviving group would be of interest to demonstrate a sustained benefit of SAP application.

Finally, evaluating the esophageal diameter using an open biopsy forceps might be imprecise and subjective. For this reason, we performed barium esophagogram that provided consistent data with endoscopic measurements.

In conclusion, the application of a self-assembling peptide matrix on a circumferential esophageal ESD wound significantly reduced the occurrence of symptomatic stricture at day 14 in our animal model. This SAP matrix, already approved for Human use for surgical and endoscopic hemostasis, allowed a quick, easy and safe application.
